# Foundations for Meaningful Consent in Canada’s Digital Health Ecosystem: Retrospective Study

**DOI:** 10.2196/30986

**Published:** 2022-03-31

**Authors:** Nelson Shen, Iman Kassam, Haoyu Zhao, Sheng Chen, Wei Wang, Sarah Wickham, Gillian Strudwick, Abigail Carter-Langford

**Affiliations:** 1 Centre for Complex Interventions Centre for Addiction and Mental Health Toronto, ON Canada; 2 Institute of Health Policy, Management and Evaluation University of Toronto Toronto, ON Canada; 3 College of Public Health University of South Florida Tampa, FL United States; 4 Canada Health Infoway Toronto, ON Canada

**Keywords:** consent, eConsent, privacy, trust, digital health, health information exchange, patient perspective, health informatics, Canada

## Abstract

**Background:**

Canadians are increasingly gaining web-based access to digital health services, and they expect to access their data from these services through a central patient access channel. Implementing data sharing between these services will require patient trust that is fostered through meaningful consent and consent management. Understanding user consent requirements and information needs is necessary for developing a trustworthy and transparent consent management system.

**Objective:**

The objective of this study is to explore consent management preferences and information needs to support meaningful consent.

**Methods:**

A secondary analysis of a national survey was conducted using a retrospective descriptive study design. The 2019 cross-sectional survey used a series of vignettes and consent scenarios to explore Canadians’ privacy perspectives and preferences regarding consent management. Nonparametric tests and logistic regression analyses were conducted to identify the differences and associations between various factors.

**Results:**

Of the 1017 total responses, 716 (70.4%) participants self-identified as potential users. Of the potential users, almost all (672/716, 93.8%) felt that the ability to control their data was important, whereas some (385/716, 53.8%) believed that an *all or none* control at the data source level was adequate. Most potential users preferred new data sources to be accessible by health care providers (546/716, 76.3%) and delegated parties (389/716, 54.3%) by default. Prior digital health use was associated with greater odds of granting default access when compared with no prior use, with the greatest odds of granting default access to digital health service providers (odds ratio 2.17, 95% CI 1.36-3.46). From a list of 9 information elements found in consent forms, potential users selected an average of 5.64 (SD 2.68) and 5.54 (SD 2.85) items to feel informed in consenting to data access by care partners and commercial digital health service providers, respectively. There was no significant difference in the number of items selected between the 2 scenarios (*P*>.05); however, there were significant differences (*P*<.05) in information types that were selected between the scenarios.

**Conclusions:**

A majority of survey participants reported that they would register and use a patient access channel and believed that the ability to control data access was important, especially as it pertains to access by those outside their care. These findings suggest that a broad *all or none* approach based on data source may be accepted; however, approximately one-fifth of potential users were unable to decide. Although vignettes were used to introduce the questions, this study showed that more context is required for potential users to make informed consent decisions. Understanding their information needs will be critical, as these needs vary with the use case, highlighting the importance of prioritizing and tailoring information to enable meaningful consent.

## Introduction

### Background

Canadians are becoming increasingly aware of digital health tools and services to support their health and wellness and are beginning to demand that they have greater access to their data that are held within these tools and services. Those who accessed their health records reported that they were more knowledgeable, informed, and confident about the care they received [1,2]. Although there are benefits to having a wide variety of digital health tools and services available, the rapid growth of the digital health ecosystem has resulted in silos of patient data. The prospect of universally connecting digital health tools, such as patient portals, is a challenge, given the large number of data exchange protocols required to share information between all points in a patient’s journey [3]. Historically, patient portals have been implemented at the organizational level and tethered to their organizational electronic health record (EHR) system. As these portals seldom exchange information between organizations, patients may end up with multiple portals of siloed data based on the various points where they seek care [4]. As a result, many patients have fragmented, limited, or no electronic access to their personal health information (PHI), giving patients an incomplete picture of their overall health to support their health care decisions. Furthermore, the multiplicity of tools and services may provide an additional burden to patients as they will need to manage the different log-ins and privacy preferences for each one.

There are growing patient demands and expectations for web-based access to their consolidated clinical and self-generated data through a single access point, recognizing that it will *make their lives better* [5]. A patient access channel serves as a trusted access point, granting patients authenticated access to their PHI and digital services data within a single platform. This allows patients to manage the collection, use, and disclosure of their PHI. Patients have the right to control how their information is collected and used, which is the definition of information privacy [6]. Canadian legislative frameworks provide protection and, generally, enable individuals to limit the use and disclosure of their records to certain individuals for specific purposes [7]. Implementing a consent management system would empower users to exercise their data-sharing preferences [8,9].

### Privacy Notices and Consent

Canadian legislation also requires consent for the collection, use, and disclosure of personal information and PHI; however, consent is seldom transparent or informed, leaving patients unaware of how their data are used and with minimal control over their data [10]. Given the largely unregulated commercial digital health ecosystem, digital health services are founded in a business model where user data are often sold for marketing or other purposes that the user may not be able to understand or foresee [11,12]. In these contexts, consent is illusory and a form of *coercion* as it does not reflect informed choice—individuals are left with the ultimatum to use or not with minimal understanding of what they are consenting to [13]. On average, privacy notices are 3964 words in length and take 18 minutes to read [14]; moreover, they are written at a postuniversity level [15]. There is an ethical imperative to improve the transparency of data use and user control of data to avoid any future exploitation by entities collecting the data [16,17].

The patient access channel offers the potential to implement consent standards that enable transparent and meaningful consent. The Office of the Privacy Commissioner of Canada Meaningful Consent Guidelines include actionable recommendations for organizations to strengthen their digital consent practices [18] by:

1. Emphasizing key elements
2. Allowing individuals to control the level of detail they get and when
3. Providing individuals with clear options to say *yes* or *no*
4. Being innovative and creative
5. Considering the consumer’s perspective
6. Making consent a dynamic and ongoing process
7. Being accountable and standing ready to demonstrate compliance

Although Meaningful Consent Guidelines provide a set of heuristics to improve consent processes, they are not specific to the digital health context [18]; moreover, they are only recommendations and do not require vendor compliance. The success of digital health requires trust and transparency in data use [19-21]. With privacy and trust as 2 intertwined antecedents to technology use and data-sharing behaviors [22], where their absence negatively affects use and behaviors, it is critical to understand the patient’s expectations of privacy to foster trust, acceptance, and use.

### Objective

A 2-stage stakeholder engagement project was conducted by Canada Health Infoway to explore the user consent requirements of a patient access channel and the privacy considerations of its implementation. It consisted of a pan-Canadian survey and regional stakeholder workshops across Canada [23]. The study reported here is a retrospective analysis of the survey data. The objective of this retrospective study is to provide a more granular understanding of user preferences for consent management.

## Methods

### Study Design

This retrospective study uses data from a cross-sectional national web-based survey conducted between October 2 and October 15, 2019, by Canada Health Infoway. This study explored how consent management preferences and information needs differ across various patient characteristics. Specifically, this study asked the following research questions (RQs):

- RQ1: What are the data control and consent management preferences of potential patient access channel users?
- RQ2: How do information needs differ among individuals when making an informed decision to share their health data with different individuals or entities?

### Data Collection

The survey comprised a series of hypothetical vignettes and consent scenarios to solicit participants’ perspectives on the consent management service and its functionalities through a mix of closed-and open-ended questions (see Multimedia Appendix 1 for the detailed vignettes and consent scenarios). There were four sections to the survey: (1) participant characteristics, (2) intention to register for the consent management service, (3) consent management use case scenarios, and (4) demographics.

The survey was administered electronically by a Canadian marketing research firm (Leger Marketing) to its pan-Canadian web panel. Using their pan-Canadian web panel, a 20-minute web-based survey was administered to the general Canadian population, reaching across the 10 provinces. The sampling strategy focused on potential digital health service users (ie, those with frequent interactions with the health care system) and used a proportional quota sampling strategy to recruit equal proportions of adults and older adults, with quotas set at 50% for adults and 80% for older adults with at least one chronic condition. The surveys were made available in English and French. Participants were eligible to participate in the survey if they were Canadian citizens, aged ≥18 years, currently live in Canada, and were within the provincial quotas for adults and seniors with chronic conditions. The survey had a view rate of 16.67% (1666/9997) and a completion rate of 61.04% (1017/1666).

### Measures

#### Overview

This study’s analytic frame comprises potential users of the patient access channel. Potential users were defined as participants who indicated that they would register to use a hypothetical patient access channel in the first set of vignettes. The vignette presented information about Canada Health Infoway and the functionality of the patient access channel (or *gateway*). Participants were then asked how likely they would register for the gateway using a 4-point Likert scale (ranging from not at all likely to very likely). Participants were also provided with an *I don’t know* option throughout the survey. The second vignette introduced a *trust framework* as the *rules of operation and participation, such as policies and agreements around data sharing and how users can control their health information*. It also presents information on consent management, single sign-on, and privacy safeguards. Participants were then asked how likely it was that they would register for the gateway based on their understanding of the trust framework and the availability of safeguards. Participants who answered *somewhat* or *very* likely were categorized as potential users.

User characteristics (ie, demographics and user experiences) were used as covariates in the analysis. The variables that exhibited a low frequency of response for some scale points were collapsed into categories to improve the statistical power of the analysis [24]. Sociodemographic data included sex (male and female), age (18-44 years, 45-64 years, and ≥65 years), income (>CAD $80,000 [US $62,380] and <CAD $80,000 [US $62,380]), and region (Atlantic, Central, Prairies, and West Coast). User experiences comprised health care use (high users or low users), patient engagement (engaged or not engaged), digital health user (user or nonuser), perceived quality of care (good or poor), past web-based experiences (good or poor), health care privacy experiences (good or poor), past privacy breaches (no past breach, breach resolved, or breach not resolved), perceived confidentiality of PHI (private or not private), perceived sensitivity of PHI (high sensitivity or low sensitivity), and perceived sensitivity of digital health data (high sensitivity or low sensitivity). A median cutoff was used to establish the threshold for perceived sensitivity variables as the categories had no theoretical grounding or frame of reference. Further details about the outcome variables and covariates can be found in Multimedia Appendix 2. The full survey can be found in Multimedia Appendix 3.

There are four variables of interest in this study: (1) the importance of consent management, (2) adequacy of broad consent, (3) entities with default access to user data, and (4) user information needs to make an informed decision about data sharing.

#### Consent Management Preferences

Participants were presented with a vignette about privacy controls and the gateway function of enabling consent directives to block or restrict access to their PHI. Participants were then asked to rate the *importance of having the ability to change privacy preferences for sharing PHI* on a 4-point ordinal scale (*not at all important* to *very important*).

The next vignette presented a scenario regarding broad consent, where a data recipient would receive either *all or none* of a particular data source (eg, medical history, laboratory records, clinical and diagnostics, and e-service data). Participants were asked to assess whether the broad access control reflected their needs or did not reflect their needs or if they did not know.

For default access, participants were presented with a scenario where they enrolled in a new digital health service and were asked to select the entities to whom they would grant default access to new sources of data. Given that they still had the ability to apply consent directives, they were asked to select the following entities to whom they would grant default access to the new source of information (ie, select all that apply): health care providers, authorized members (ie, family and friends), digital health services and tools, or none of the above (ie, grant access individually or to each group).

#### Information Needs

To assess user information needs for informed consent, participants were first presented a vignette on consent management, which outlined the types of PHI they may access in the gateway and introduced an access control function that allows patients to authorize access to their PHI to health care providers, family and friends, and digital service vendors. Participants were asked to select the types of information they required to make an informed decision on whether to share their data in two scenarios: sharing with friends and family (scenario 1) and sharing with digital health providers for the digital health service (scenario 2).

Participants were provided with a list of information types that are found on consent forms and privacy notices and were asked to select all that applied. The nine information types were as follows: what types of information that the digital service can access, what the digital service can do with their data, potential risks and benefits of granting access, how to ask more questions about information sharing or privacy, how to file complaints about how information is shared, functions that allow them to monitor activity, types of data access controls available, and how to revoke access.

### Data Analysis

First, the frequencies and percentages of the characteristics and demographics of all potential users were reported. For RQ1, frequencies for the importance of access control, adequacy of *all or none* access control based on data source, and default access to PHI were shown. Logistic regression was applied to evaluate the factors associated with the adequacy of access control, whether knowing it met their needs regarding adequacy, and granting default access. In the model-building procedure, a small subset of participants was excluded from the total sample because of the limited number of observations within each cell. The number of participants and the corresponding percentages were reported for the frequency analysis. Adjusted odds ratios (ORs) and 95% CIs were reported for logistic regression results.

For RQ2, the Friedman test was used to assess the difference in the number of items selected between the 2 scenarios in terms of sharing their information. The McNemar test was also performed to check if the frequency of each item differed between the 2 scenarios. All statistical analyses were conducted using SAS software (SAS Enterprise Guide 7.1; SAS Institute Inc).

### Ethics Approval

This study was approved by the Research and Ethics Board at the Centre for Addiction and Mental Health (REB#114/2020) in Toronto, Canada.

## Results

### Overall Results

Of the 1017 responses, 716 (70.4%) *potential users* of the patient access channel were identified. The potential user characteristics can be found in Table 1. Over three-quarters were low service users (559/716, 78.1%), noncaregivers (621/716, 86.7%), engaged patients (612/716, 85.5%), and satisfied with their quality of care (609/716, 85.1%). Over half had used digital health tools previously (471/716, 65.8%) and rated their PHI (364/716, 50.8%) and digital health data as sensitive (423/716, 59.1%). Most potential users reported having positive privacy experiences on the web (535/716, 74.7%), positive health care privacy experiences (643/716, 89.8%), and trust in the confidentiality of their records in the health care system (644/716, 89.9%). The final sample size of potential users for the logistic regression model was 712.

**Table 1:** Characteristics of potential users (N=716).

Characteristic			Values, n (%)
**Sex**			
	Female	343 (47.9)	
	Male	369 (51.5)	
	Transgender^a^	2 (0.3)	
	Other^a^	1 (0.1)	
	PNA^a,b^	1 (0.1)	
**Age (years)**			
	18-44	204 (28.5)	
	45-64	155 (21.7)	
	≥65	357 (49.9)	
**Region**			
	Atlantic	47 (6.6)	
	Central	418 (58.4)	
	Prairie	145 (20.3)	
	West Coast	106 (14.8)	
**Income (CAD$; US $)**			
	<$80,000 ($62,380)	401 (56)	
	>$80,000 ($62,380)	258 (36)	
	PNA	57 (8)	
**Health care use**			
	High (>20)	146 (20.4)	
	Low (≤20)	559 (78.1)	
	IDK^c^	11 (1.5)	
**Caregiver**			
	No	621 (86.7)	
	Yes	95 (13.3)	
**Quality of care**			
	Good	609 (85.1)	
	Poor	92 (12.9)	
	IDK	15 (2.1)	
**Prior digital health use**			
	Yes	471 (65.8)	
	No	245 (34.2)	
**Engaged patient**			
	Yes	612 (85.5)	
	No	104 (14.5)	
**Sensitivity of PHI^d^**			
	High (≥10)	364 (50.8)	
	Low (<10)	352 (49.2)	
**Sensitivity of digital health data**			
	High (≥11)	383 (53.5)	
	Low (<11)	333 (46.5)	
**Web-based privacy experience**			
	Good	535 (74.7)	
	Poor	134 (18.7)	
	IDK	47 (6.6)	
**Privacy breach**			
	Yes, resolved	70 (9.8)	
	Yes, not resolved or IDK	29 (4.1)	
	No breach	617 (86.2)	
**Health care privacy experiences**			
	Good	643 (89.8)	
	Poor	51 (7.1)	
	IDK	22 (3.1)	
**Confidentiality of records**			
	Private	644 (89.9)	
	Not private	37 (5.2)	
	IDK	35 (4.9)	

^a^Indicates subpopulations that were excluded from the logistic regression model.

^b^PNA: prefer not to answer.

^c^IDK: I do not know.

^d^PHI: personal health information.

### RQ1: What Are the Data Control and Consent Management Preferences of Potential Patient Access Channel Users?

#### Importance of Access Control

Overall, 93.8% (672/716) of the potential users believed it was important (126/716, 18%) or very important (543/716, 75.8%) to have the ability to control their privacy preferences. Further subanalyses were not conducted as the distribution of responses would not allow for the detection of differences between the options.

#### Adequacy of All or None Access Control Based on Data Source

Approximately 53.8% (385/716) of the potential users felt that an *all or none* approach based on the data source to control data access was adequate for their needs, whereas 29.2% (209/716) did not, and 17.0% (122/716) did not know.

Geographic location and income were the only factors that were significantly associated with *all or none* being adequate for the participant’s needs. Potential users from the Prairies were 50% less likely than those from Central Canada to feel that it was adequate (OR 0.50, 95% CI 0.32-0.78). Potential users earning >CAD $80,000 (US $62,380) or potential users that did not disclose their income were 42% and 68% less likely to find *all or none* adequate than low-income earners (<CAD $80,000 [US $62,380]; OR 0.58, 95% CI 0.40-0.86; OR 0.32, 95% CI 0.16-0.66). Potential users with high income were 129% more likely to know that an *all or none* approach would meet their needs than those with low income (OR 2.29, 95% CI 1.36-3.83). Those who used digital health tools previously were associated with a 109% increased likelihood to know that an *all or none* approach would meet their needs than those who did not (OR 2.09, 95% CI 1.34-3.25). The results of the logistic regression analysis can be found in Table 2.

**Table 2:** Comparison of the adequacy of all or none based on participant characteristics.

Characteristics		Adequate for needs, odds ratio (95% CI)	Know versus not know, odds ratio (95% CI)
**Sex**			
	Male	Reference	Reference
	Female	0.77 (0.53-1.13)	0.72 (0.47-1.11)
**Age (years)**			
	18-45	Reference	Reference
	46-64	0.80 (0.48-1.34)	1.09 (0.55-2.15)
	≥65	0.74 (0.47-1.17)	0.68 (0.39-1.17)
**Health care use**			
	Low	Reference	Reference
	High	1.33 (0.83-2.13)	0.67 (0.40-1.12)
	IDK^a^	0.99 (0.17-5.58)	0.38 (0.09-1.62)
**Region**			
	Central	Reference	Reference
	Atlantic	0.54 (0.26-1.10)	1.45 (0.56-3.77)
	Prairie	0.50 (0.32-0.78)^b^	0.88 (0.52-1.50)
	West Coast	0.74 (0.43-1.25)	0.59 (0.34-1.05)
**Caregiver**			
	No	Reference	Reference
	Yes	1.60 (0.93-2.74)	2.00 (0.93-4.30)
**Income (CAD $; US $)**			
	<$80,000 ($62,380)	Reference	Reference
	>$80,000 ($62,380)	0.58 (0.40-0.86)^b^	2.29 (1.36-3.83)^b^
	PNA^c^	0.32 (0.16-0.66)^b^	0.79 (0.40-1.55)
**Engaged patient**			
	No	Reference	Reference
	Yes	0.92 (0.53-1.61)	0.86 (0.44-1.69)
**Quality of care**			
	Poor	Reference	Reference
	Good	0.92 (0.49-1.76)	1.45 (0.73-2.87)
	IDK	2.18 (0.38-12.56)	1.26 (0.27-5.99)
**Prior digital health use**			
	No	Reference	Reference
	Yes	0.75 (0.50-1.13)	2.09 (1.34-3.25)^b^
**Sensitivity of PHI^d^**			
	Low	Reference	Reference
	High	0.68 (0.44-1.05)	1.07 (0.65-1.77)
**Sensitivity of health data**			
	Low	Reference	Reference
	High	1.25 (0.81-1.92)	0.70 (0.42-1.17)
**Web-based privacy experiences**			
	Poor	Reference	Reference
	Good	1.27 (0.77-2.08)	1.18 (0.66-2.11)
	IDK	1.28 (0.50-3.27)	0.52 (0.21-1.25)
**Past privacy breach**			
	Not resolved or IDK	Reference	Reference
	Resolved	1.22 (0.44-3.40)	1.66 (0.43-6.42)
	No breaches	1.53 (0.63-3.74)	0.97 (0.32-2.97)
**Health care privacy experiences**			
	Poor	Reference	Reference
	Good	1.73 (0.81-3.71)	0.54 (0.20-1.48)
	IDK	0.64 (0.13-3.12)	0.27 (0.06-1.19)
**Confidentiality of PHI**			
	Not private	Reference	Reference
	Private	0.95 (0.38-2.35)	1.09 (0.40-2.92)
	IDK	1.90 (0.50-7.27)	0.87 (0.25-2.99)

^a^IDK: I do not know.

^b^Signifies a significant association when compared with the reference group.

^c^PNA: prefer not to answer.

^d^PHI: personal health information.

#### Default Access to PHI

Most potential users would grant default access to new data that become available to their health care providers (546/716, 76.3%) or authorized members, such as family, friends, and other care partners (389/716, 54.3%). Approximately one-fifth would grant default access to their digital health service provider for use with digital health services (138/716, 19.3%). Finally, 14.8% (106/716) of the potential users would not grant default access to anyone. Factors associated with granting default access were prior digital health use, health care privacy experiences, caregiver status, sex, and perceived sensitivity of PHI (Table 3).

Prior use of digital health tools was associated with a greater likelihood of granting default access to the 3 entities as there was a 66% greater likelihood of granting default access to health care providers (OR 1.66, 95% CI 1.14-2.44), 101% greater likelihood of granting default access to authorized members (OR 2.01, 95% CI 1.43-2.81), and 117% greater likelihood of granting default access to digital health service providers (OR 2.17, 95% CI 1.36-3.46). Those with prior digital health tool use were 53% less likely to not want to grant default access to anyone (OR 0.47, 95% CI 0.29-0.74). Those with positive health care privacy experiences were 156% more likely to grant default access to health care providers (OR 2.56, 95% CI 1.24-5.29) and 70% less likely to not grant default access than those with poor experiences (OR 0.30, 95% CI 0.20-0.70).

Service providers were 142% more likely to gain default access from caregivers (OR 2.42, 95% CI 1.45-4.04) but 39% less likely to gain default access from females (OR 0.61, 95% CI 0.40-0.92). Authorized users were 34% less likely to gain default access (OR 0.66, 95% CI 0.46-0.96) from potential users who had high perceived PHI sensitivity in comparison with those with low perceived PHI sensitivity. Those with high PHI sensitivity were also 126% more likely to not grant default access to anyone (OR 2.26, 95% CI 1.30-3.93) than those with low perceived PHI sensitivity.

**Table 3:** Comparison of default access based on participant characteristics.

Characteristics			Odds ratio (95% CI)						
			HCP^a^		Authorized members		DHSP^b^		No one
**Sex**									
	Male	Reference		Reference		Reference		Reference	
	Female	1.12 (0.77-1.63)		0.94 (0.68-1.29)		0.61 (0.40-0.92)^c^		0.90 (0.57-1.41)	
**Age (years)**									
	18-44	Reference		Reference		Reference		Reference	
	45-64	0.85 (0.51-1.43)		1.31 (0.84-2.06)		0.94 (0.54-1.64)		1.25 (0.65-2.39)	
	≥65	0.87 (0.55-1.37)		1.35 (0.92-1.99)		0.90 (0.55-1.47)		1.39 (0.78-2.47)	
**Health care use**									
	Low (≤20)	Reference		Reference		Reference		Reference	
	High (>20)	1.33 (0.82-2.17)		0.79 (0.54-1.18)		0.99 (0.60-1.63)		1.04 (0.58-1.85)	
	IDK^d^	0.43 (0.12-1.55)		0.81 (0.22-2.96)		0.43 (0.05-3.73)		2.16 (0.48-9.73)	
**Region**									
	Central	Reference		Reference		Reference		Reference	
	Atlantic	0.85 (0.41-1.73)		1.13 (0.60-2.15)		1.38 (0.62-3.07)		0.98 (0.41-2.35)	
	Prairie	1.31 (0.80-2.15)		0.72 (0.48-1.06)		0.72 (0.42-1.23)		0.61 (0.32-1.16)	
	West Coast	0.72 (0.44-1.18)		0.82 (0.52-1.28)		0.93 (0.52-1.65)		1.68 (0.95-2.97)	
**Caregiver**									
	No	Reference		Reference		Reference		Reference	
	Yes	0.75 (0.45-1.25)		1.42 (0.89-2.27)		2.42 (1.45-4.04)^c^		0.56 (0.26-1.19)	
**Income (CAD $; US $)**									
	<$80,000 ($62,380)	Reference		Reference		Reference		Reference	
	≥$80,000 ($62,380)	0.89 (0.60-1.31)		1.23 (0.88-1.72)		1.26 (0.83-1.91)		1.20 (0.74-1.95)	
	PNA^e^	0.73 (0.38-1.40)		0.75 (0.42-1.34)		0.65 (0.26-1.63)		1.77 (0.86-3.68)	
**Engaged patient**									
	No	Reference		Reference		Reference		Reference	
	Yes	0.95 (0.54-1.66)		1.14 (0.70-1.84)		0.63 (0.35-1.14)		1.29 (0.63-2.65)	
**Quality of care**									
	Poor	Reference		Reference		Reference		Reference	
	Good	1.07 (0.58-1.98)		0.87 (0.51-1.49)		0.75 (0.39-1.46)		0.79 (0.38-1.65)	
	IDK	1.27 (0.29-5.59)		0.33 (0.09-1.16)		1.54 (0.36-6.56)		0.75 (0.12-4.58)	
**Prior digital health use**									
	No	Reference		Reference		Reference		Reference	
	Yes	1.66 (1.14-2.44)^c^		2.01 (1.43-2.81)^c^		2.17 (1.36-3.46)^c^		0.47 (0.29-0.74)^c^	
**Sensitivity of PHI^f^**									
	Low (<10)	Reference		Reference		Reference		Reference	
	High (≥10)	0.93 (0.60-1.43)		0.66 (0.46-0.96)^c^		1.04 (0.65-1.69)		2.26 (1.30-3.93)^c^	
**Sensitivity of health data**									
	Low (<11)	Reference		Reference		Reference		Reference	
	High (≥11)	0.91 (0.59-1.40)		1.16 (0.80-1.68)		1.13 (0.70-1.83)		0.87 (0.51-1.49)	
**Web-based privacy experiences**									
	Poor	Reference		Reference		Reference		Reference	
	Good	0.79 (0.47-1.33)		1.03 (0.66-1.59)		1.29 (0.73-2.27)		1.42 (0.73-2.75)	
	IDK	0.55 (0.23-1.29)		0.74 (0.35-1.58)		0.22 (0.04-1.07)		2.48 (0.91-6.74)	
**Past privacy breach**									
	Not resolved or IDK	Reference		Reference		Reference		Reference	
	Resolved	1.15 (0.38-3.43)		1.24 (0.49-3.11)		2.23 (0.68-7.32)		0.57 (0.15-2.13)	
	No breaches	0.91 (0.36-2.31)		0.91 (0.41-2.01)		1.30 (0.45-3.79)		0.82 (0.28-2.34)	
**Health care privacy experiences**									
	Poor	Reference		Reference		Reference		Reference	
	Good	2.56 (1.24-5.29)^c^		1.46 (0.73-2.90)		1.90 (0.67-5.35)		0.30 (0.20-0.70)^c^	
	IDK	3.75 (0.97-14.51)		1.81 (0.53-6.19)		1.48 (0.20-10.85)		0.31 (0.07-1.44)	
**Confidentiality of PHI**									
	Not private	Reference		Reference		Reference		Reference	
	Private	1.25 (0.55-2.83)		0.88 (0.41-1.87)		1.50 (0.52-4.39)		1.40 (0.48-4.14)	
	IDK	0.79 (0.26-2.38)		1.52 (0.54-4.33)		2.14 (0.49-9.32)		2.11 (0.54-8.28)	

^a^HCP: health care provider.

^b^DHSP: digital health service provider.

^c^Signifies a significant association when compared with the reference group.

^d^IDK: I do not know.

^e^PNA: prefer not to answer.

^f^PHI: personal health information.

### RQ 2: Do Information Needs Differ Among Individuals When Making an Informed Decision to Share Their Health Data With Different Individuals or Entities?

Overall, 81.8% (586/716) of potential users considered sharing their data in both scenarios (ie, potential users who did not select *I do not intend on sharing information with them*). In scenario 1, 89.3% (639/716) of potential users considered granting access to their friends and families and required an average of 5.64 (SD 2.68) of the 9 presented information types to make that decision. In scenario 2, 85.2% (610/716) of potential users considered granting commercial service providers access to their data and required an average of 5.54 (SD 2.85) of the 9 presented information types to make that decision.

**Figure 1:**
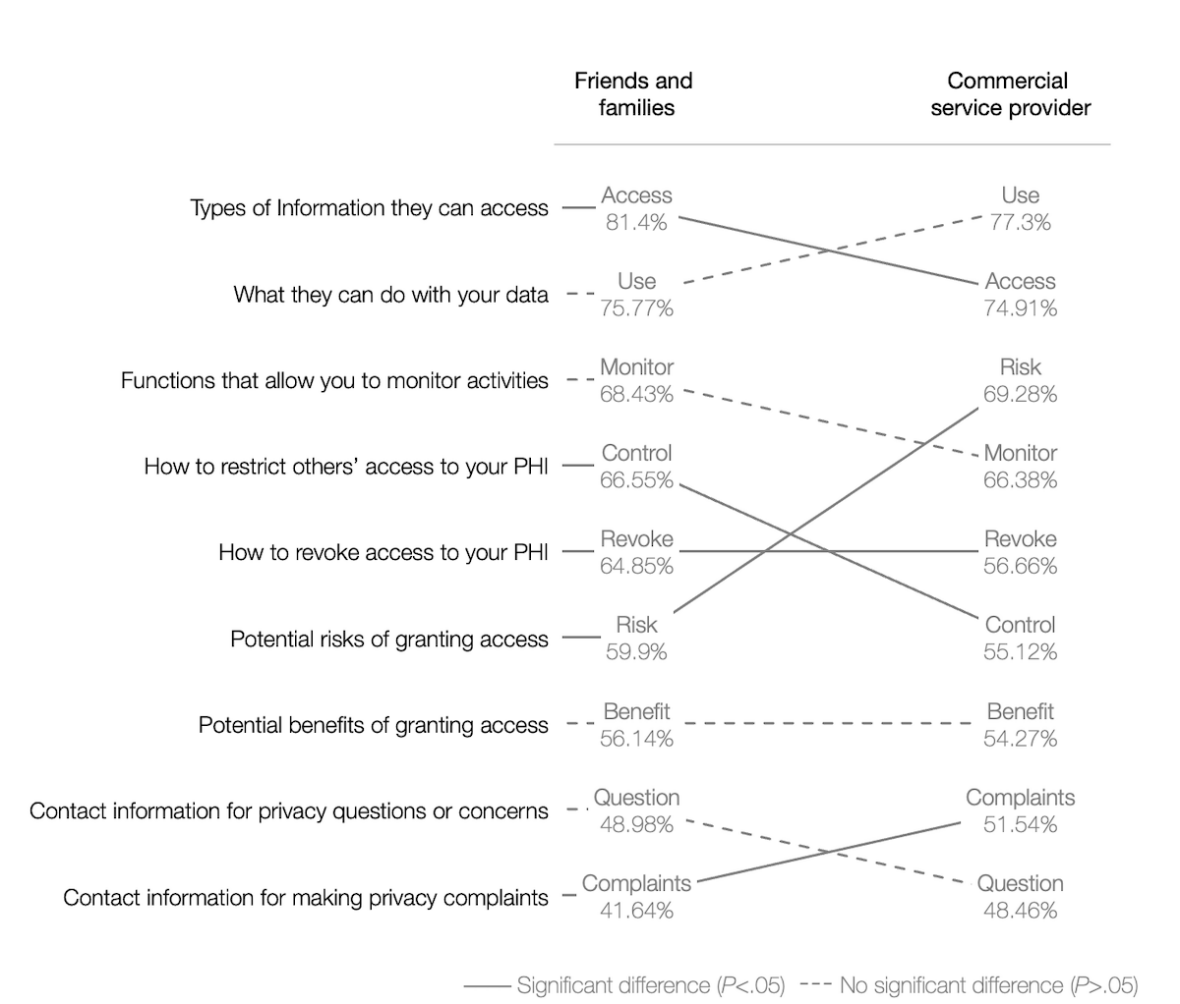
Differences in information needs required to support decisions on data sharing with friends and family and commercial service providers (ranked by frequency selected; n=586). PHI: personal health information.

There was no significant difference in the average number of information types required between the 2 scenarios (*P*>.05) for potential users who considered sharing in both scenarios (586/716, 81.8%). On the basis of the frequency of selection by this subset of potential users, the ranking of the types of information differed in the 2 scenarios (Figure 1); however, there was only a significant difference in frequency for 5 of the information types (*P*<.05). Information about accessible data types, restricting access, and revoking was selected more frequently in scenario 1. Information about potential risks and filing complaints was selected more frequently in scenario 2.

## Discussion

### Principal Findings

As society becomes increasingly interconnected, there is a corresponding patient anticipation that their PHI and digital health data can be centrally accessed through innovations such as patient access channels, all with the belief that they will make life better [5]. A core requirement critical to the adoption of these patient access channels is a consent management system, as almost all potential users value the ability to control who can access their data. This exploratory study generated some insights to consider when implementing a consent management system. First, there may be acceptance of a believed, broad *all or none* access control model by data source, as 53.8% (385/716) of potential users believed it was adequate for their needs, and 17% (122/716) were unsure. Second, the willingness to provide others with default access to PHI and data varied depending on the recipient. Finally, potential users required an average of approximately 6 key types of information to provide informed decisions regarding data sharing; however, the required types of information varied depending on the recipient. The 3 insights are discussed in detail in the following sections.

### Data Control and Consent Management Practices

Given the complexity of implementing interoperable access control in Canada [23], a broad *all or none* access control at the level of the data source may be the only option in the interim for patient access channels, especially as new data sources continuously emerge [8]. If implemented within a context of a *trust framework* in this scenario, there may be an acceptance of broad access control as over half of the potential users believed it was adequate for their needs. This finding echoes that of Grando et al [25], where broad access control was adequate for 58% of their study participants; moreover, their study was set in the context of behavioral health—an area where PHI is often perceived as more sensitive. Similarly, and surprisingly, user perceptions of the sensitivity of PHI and digital health data were not associated with adequacy, especially as data sensitivity is commonly associated with wanting greater degrees of access control because of privacy concerns [26,27]. A possible explanation is that the sample had a high level of trust in the confidentiality of their PHI and had positive dispositions about their web-based and health care privacy. This is consistent with an emerging set of evidence showing that positive perceptions of health care, trust in health care providers, and positive past privacy experiences may result in individuals having favorable views on sharing data [28-34]. Although these studies are contrary to prior findings of patients wanting more granular control options [25,27,35], their hypothetical and exploratory nature is subject to the privacy paradox [36]—the disconnect between intentions based on privacy concerns and actual behaviors. For instance, Schwartz et al [37] provided 108 patients with the ability to restrict access to their sensitive EHR data and found that 57% provided access to all listed providers and all PHI in their EHR, and 8.6% limited access by data type to specific providers. A significant minority of participants (43%) limited access to at least one provider.

Approximately one-fifth of the potential users did not know whether broad access control would be adequate, highlighting the need to better support their decision-making. The technical aspects of sharing data may be complex and may require greater literacy to appreciate the impact of broad access control [23]. This may explain why digital health use was associated with a 109% increase in the likelihood of knowing whether it is adequate. Familiarity and experience with digital health may provide individuals with heuristics to make decisions [34]. Studies show that broad access control and consent models may be acceptable when there is transparency [28,38,39] and assurance in oversight [40] regarding how the data are used. Biobank studies have shown that there are no significant differences in the willingness to share data between various consent scenarios when participants are provided with specific information on the data that are being used [38] or if there is assurance that a governing body provides oversight on how data are being used [40]. These findings can be applied to the digital health context, as a recent survey found that 80% of Canadians are willing to share their anonymized health information as long as the privacy and security of their PHI are assured [41].

Income and region were the only demographics found to be associated with adequacy perspectives on broad access control and knowing whether broad access control was adequate. Although the association between privacy attitudes and income echoes some privacy studies in health informatics, there have often been conflicting results across studies [33]. Historically, privacy research has focused on demographic variables as predictors of privacy attitudes and behaviors; however, collective evidence signals that individual demographic variables play a minor role and provide limited insight into understanding a phenomenon [33,42]. These findings are intended to inform further explorations to support implementation decisions. For instance, there may be value in understanding the underlying factors associated with those with high incomes that support their views of inadequacy and why they are more likely to know whether broad access control reflects their needs. In terms of region, health care in Canada is administered at the provincial level, where there are variations in legislation, policies, and digital health initiatives. Only a few provinces in Canada have a centralized patient portal, and the Prairie provinces of Alberta and Saskatchewan were launching theirs at the time of this study [43-45]. Understanding how these initiatives may have affected attitudes on broad access control adequacy may inform strategies on how to improve public favorability toward broad access control.

Potential users were most willing to grant default access to their health care providers, especially those with positive health care privacy experiences. Their willingness decreased as the data recipient was further removed from the point of care. Patients generally trust their physicians and those in their circle of care to keep their data confidential; however, this trust to maintain confidentiality diminishes as the data recipient is further away from those providing care (eg, health department, researchers, and corporations) [25,27,35,46]. Canadians are generally comfortable with the sharing of their PHI through EHRs with other health care providers as they believe timely and easy access to PHI is necessary for high-quality care [47], highlighting the role of contextual relevance and issue involvement [48] in data-sharing behaviors. This assertion is supported by the finding that those with prior digital health use were 66% to 117% more likely to grant default access (entity dependent) than those who did not use digital health tools. These users may have a greater stake in using digital health tools, familiarity, and perceived benefits of sharing digital health data [28-34]. Contextual relevance also mattered for users with higher perceived sensitivity of PHI, as 126% were more likely to not give anyone default access and were less likely to grant default access to family, friends, and other supporters. These individuals may not be comfortable with default access and may want more control over how new information is shared. Sharing may depend on the purpose and whether it is a necessity; moreover, these individuals may want more control over how certain information is disclosed to close social associates as it may affect their relationships. They may want to share about it in person rather than have others find it out by default through technology [34].

The value of data sharing with digital health service providers may not be as clear as there is limited trust in service providers, especially commercial vendors [19,47]. In this study, one-fifth of potential users were willing to grant default access to service providers, of whom users with prior digital health experience and caregivers were more likely to share their data. As discussed earlier, these users may have greater perceived benefits of granting default access to data to service providers [28-34]. For caregivers, sharing data for this population may be perceived to improve the tasks and stressors associated with their caregiving roles through the development of better or improved digital health services [49]. Further understanding the rationales of those trusting and skeptical of commercial service providers will be a necessity as these providers are a growing contributor to the number and types of services provided and an important source of data for patient access channels. This understanding can inform the permitted uses outlined in the trust framework and enable informed and meaningful consent.

### Information Needs

This study builds upon the Meaningful Consent Guidelines for use in the digital health context. The guidelines recommend that digital vendors emphasize four key information elements: what is collected, who has access, purpose of data collection, and potential risks. However, this study found that potential users may need 5 to 6 emphasized information elements. The additional elements of emphasis include information on monitoring access, restricting access, and revoking consent. The findings also suggest that there is a need to tailor the order of emphasized elements as they will vary depending on who is accessing the data.

This study also highlights the importance of patient engagement in ensuring that the design of consent is based on user needs rather than assumptions. For instance, presenting this consent information in clear, concise, and plain language has been advocated but seldom practiced; however, implementing this assumption is only a part of the solution. A study found that an easier to read, concise consent form neither hindered nor improved comprehension or satisfaction with the consent process among their participants [50]. In contrast, providing users with ways of customizing their experiences and consuming information is more effective [51]. User experience is an overlooked aspect that should be considered when implementing informed and meaningful consent [23,52]. To empower users to make informed choices, meaningful consent for patient access channels should be iteratively co-designed with its users to ensure that they meet their needs rather than their assumed wants [53].

### Limitations

This study provides preliminary insights to support future patient engagement in co-designing a consent management system and meaningful consent. However, these exploratory findings are not intended to be generalizable as there are limitations to consider. This study is a secondary analysis of a cross-sectional survey, providing a snapshot of a time point where perspectives may vary over time. This study relied on a series of vignettes to preface the questions. Multiple rounds of revisions were made with Canada Health Infoway’s communications department and the market research firm to improve clarity of complex concepts (eg, privacy, consent, and data sharing). Prompts with these concepts include languages with high readability scores, which may have influenced some responses, especially those with lower digital literacy skills [54].

There are also inherent limitations to data collection through a survey panel as it only includes people who participate in the web panels managed by the company and relies on the self-selection of participants. The web-based nature of the survey may have excluded the perspectives of individuals with limited internet access. However, approximately 94% of Canadian households currently have access to the internet [55]. The purposive sampling strategy limits generalizability to the broader Canadian population as recruitment focused on frequent users of health care and excluded the Canadian territories (ie, early adopters of a patient access channel) [56,57]. The identified users in this study may be more engaged and experienced with digital health tools, thereby perceiving greater benefits and a greater willingness to share their data. The low response rate should also be considered as it may limit the diversity and nuance of perspectives because of the information lost through the combination or omission of demographics and participant characteristics for data analysis (eg, individuals who are transgender and other identifying genders). Future public and stakeholder engagement activities will require a greater in-depth investigation in co-designing consent management for patient access channels. Recognizing the ethical transgressions in trust in health care and research of marginalized and vulnerable communities [58], future research must include more diversity in perspectives to understand how to equitably strengthen meaningful consent and consent management practices.

### Conclusions

Providing patients with the ability to manage their consent and control access to their PHI is valued by potential users of a patient access channel. Following the Office of the Privacy Commissioner of Canada’s Meaningful Consent Guidelines, future work should continue to *consider the consumer’s perspective* by involving them throughout the development and implementation processes [18]. Given technological limitations, future public engagement should investigate what makes broad access control acceptable and how to communicate its implications meaningfully and transparently. Future research should also focus on understanding user requirements for consent to further adapt the Meaningful Consent Guidelines for the digital health context. Understanding how to foster patient trust and how to empower them to feel confident in their data-sharing decisions is necessary for the success of patient access channels and the realization of the transformative potential of the evolving digital health ecosystem.

## Appendix

Multimedia Appendix 1 Consent scenarios and vignettes.

Multimedia Appendix 2 Covariate definitions and outcome variables for logistic regression.

Multimedia Appendix 3 Full survey questionnaire.

## Abbreviations

CIHR: Canadian Institutes of Health Research
EHR: electronic health record
OR: odds ratio
PHI: personal health information
RQ: research question
