# Supplementary material for: Foundations for Meaningful Consent in Canada’s Digital Health Ecosystem: Retrospective Study
Source: JMIR Med Inform. 2022 Mar 31;10(3):e30986. doi: 10.2196/30986 (PMC9015739; doi:10.2196/30986)
Supplement: Multimedia Appendix 1 [file medinform_v10i3e30986_app1.docx]

| **Vignette 1: Perceptions About Access Health**  **1a – About Access Health**  Canada Health Infoway is a federally funded, independent, not-for-profit  organization, who works to implement the use of digital health solutions across  Canada.  Canada Health Infoway, with funding from Health Canada, has a program called  ACCESS Health that is providing Canadians with access to their personal health information and to digitally enabled health services. The program aims to provide you as a Canadian with a greater ability to manage your care, more convenience and increased control over your personal health information.  One part of the program is a digital platform that Canada Health Infoway is building, called the ACCESS Gateway. Available to every Canadian, once built, the service will allow you do things like:   - View your personal health information online, - Share your health information with others (e.g., friends/family, health care professionals like your doctor, or commercial service providers such as health tracking/monitoring apps) - Electronically book medical appointments, - Securely communicate with care providers, and - Use self-management applications.   Canada Health Infoway is engaging with Canadians to better understand their needs, priorities, and expectations to guide the development of the ACCESS Gateway. Based on your understanding, would you register for the ACCESS Gateway?  1b – Structural Assurances  Canada Health Infoway's "Trust Framework" is the go-to guide for what participants in shared digital health systems (such as the ACCESS Health Gateway) need in order for health information systems to be able to connect and share with each other in the way that meets the needs and expectations of Canadians.  It includes rules for operation and participation, such as policies and agreements around data sharing and how users can control their health information. This allows users to access multiple systems and data sources securely and easily with fewer credentials (e.g., avoid multiple usernames and passwords), via the ACCESS Gateway.  Individuals will be able to control access to their health information (i.e., allowing access of your data to certain individuals – for example, friends/family, healthcare providers, commercial service providers) in a secure manner. This lets individuals control the access to personal health information by enabling/disabling it for various mobile apps/website applications from one place.  Based on your understanding of the “Trust Framework” and the availability of the safeguards* listed in the previous question, would you register and use the ACCESS Gateway?  **safeguards: Ability to access health records online; Ability to hide or mask sensitive information from those you have given access; Regular privacy and security audits of ACCESS Gateway; Text or email notification when a healthcare provider accesses my records; Ability to view a history of who accessed my records and when; Knowing I would be informed of any privacy and security breaches that may have occurred with my health records; More knowledge about the policy and procedures that protect my health records; A strict policy that my health information could not be sold or released to any organization/business that was not a part of the healthcare system.*  Textbox 1. Hypothetical Vignette 1 |
| --- |

| **Vignette 2: Consent Management**  **Vignette 2A- Consent Management**  The ACCESS Gateway will include consent technology that will manage individual’s preferences for sharing their health information. For example, an individual may use a healthcare application, that could be a mobile app or web application, which provides access to their personal health information. Health data may include, but is not limited to:   - patient summary (e.g., history of diagnoses, investigations, treatments, care plan) - medication history - lab results - diagnostic reports (e.g., medical imaging) - data from digital services and tools   If the healthcare application is connected to the ACCESS Gateway, an individual will be able to access their personal health information in one place through the ACCESS Gateway and put preference in place for who can access their personal health information.  Vignette 2b- Delegating access  The ACCESS Gateway may provide ways to allow patients to authorize or delegate access to their personal health information to:   - healthcare providers, - verified members (such as family and friends), - digital services and tools they have chosen to use.   You have a partner, friend, or family member who supports you in managing your health. You are considering authorizing them to access your personal health information available through ACCESS Gateway. To do so, you must provide consent to grant them access to your records.    What types of information* do you require in order to make an informed decision? *Please select all that apply.*  You are interested in enrolling in a digital service that is available from a commercial service provider (i.e. a company storing medical information that allows you to manage your health). The digital tool requires access to your lab results to allow you to track your blood work and provide you with curated content for your self-management. To use the tool, you must provide consent to grant it access to your records.  What types of information* do you require in order to make an informed decision? *Please select all that apply.*  **Information elements include: Type(s) of information the they can access; What they can do with your data; Potential risks of granting access; Potential benefits of granting access; How to ask more questions around information sharing / privacy; How to file complaints around how information is shared ; ACCESS Gateway functions that allow you to monitor activity; Types of data access controls available; How to revoke access; Other – please specify ______; None of the above; I do not intend on sharing information with them*  Vignette 2c – Privacy Control  The ACCESS Gateway will also allow individuals to securely control access to their information via consent directives (i.e. allowing or restricting access of your data to certain individuals – for example, friends/family, healthcare providers, commercial service providers).  Consent directives gives individuals the option to block or restrict access to their personal health information.  When sharing personal health information, people have control over their privacy preferences (e.g., who can see the personal health information, why they can see it, which parts they can see, etc.). How important is the ability to set and change your privacy preferences for sharing your personal health information?  If the ACCESS Gateway allows you to consent to share either “all” or “none” of the personal health information from a specific source* with those individuals or groups you granted access.  *Data sources include: medical history from clinics and hospitals; lab records; clinical and diagnostic imaging; drug and pharmacy services; lab testing, and e-services data.*  What are your feelings about this method for giving your consent to share your personal health information? *Please select one only.* Textbox 3. Hypothetical Vignette 2 |
| --- |
|  |
