# Supplementary material for: Foundations for Meaningful Consent in Canada’s Digital Health Ecosystem: Retrospective Study
Source: JMIR Med Inform. 2022 Mar 31;10(3):e30986. doi: 10.2196/30986 (PMC9015739; doi:10.2196/30986)
Supplement: Multimedia Appendix 2 [file medinform_v10i3e30986_app2.docx]

# Multimedia Appendix 2: Covariate definitions and outcome variables for logistic regression

| **Co-variates**** | **Outcome Variable** |
| --- | --- |
| - S1: Gender   - Change to sex – m or f - S2: Age (continuous – need to categorize)   - 18-44   - 45-64   - 65+ - S3b: Healthcare Utilization   - High User = 21+   - Low User = less than 20 - S4: Province   - Atlantic – NFL, PEI, NS, NB   - Central – QC, ON   - Prairie – MB, SK, AB   - West Coast - BC - D3: Are you a caregiver/friend/family member (select 1)   - 1; (2+99) - D7: Annual household income (select 1)   - ≥$80 K CAD   - <$80K CAD   - Prefer not to answer - A2 & A3 - Engaged Patients   - Yes; No   - If answer 1-2 for BOTH A2 and A3 - A4: Perceived Quality of Care   - Poor + Fair; Good + Excellent; 98 - Digitally Engaged Patients - A1a & A1b: Digital Users   - If answered 7 (other health-related purposes) for A1a AND selected any of the options except 99 (none of the above) A1b - A5a: Sensitivity of PHI   - Median Cut for High and Low Sensitivity - A5b: Sensitivity of personal health data   - Median Cut for High and Low Sensitivity - A6a: Level of online privacy - A6b + A6d: Privacy Breach   - Combine A6b with A6d; replace A6b response 1 with response 1-3 a6d - A7a: Level of Privacy in healthcare - A7d: Trust in health care providers | RQ5a – importance of access control*   - C5: Importance of controlling privacy preferences   - Important = 1 or 2   - No important = 3 or 4   RQ5b – “all or none” adequacy   - C6a: adequacy of “all or none”   - Binary 1 or 2; idk (99)   RQ5c – Default Access   - Treat each as variable – 3 different questions   - Health care provider   - Authorized member   - Digital Health Service Provider   - No-one   *No longer doing analysis for C5 b/c not meaningful (96% rated important).  **note: same covariates as RQ4 |
