# Supplementary material for: Foundations for Meaningful Consent in Canada’s Digital Health Ecosystem: Retrospective Study
Source: JMIR Med Inform. 2022 Mar 31;10(3):e30986. doi: 10.2196/30986 (PMC9015739; doi:10.2196/30986)
Supplement: Multimedia Appendix 3 [file medinform_v10i3e30986_app3.docx]

# **INFOWAY ACCESS CONSENT SCENARIO QUESTIONNAIRE**

# **DISPLAY:**

Thank you for agreeing to participate in our survey. Your responses will be held strictly confidential. **The survey will take approximately 20 minutes to complete.**

**SCREENER SECTION:**

S1. What is your gender?

| Female | 1 |
| --- | --- |
| Male | 2 |
| Transgender | 3 |
| Other | 4 |
| I prefer not to answer | 99 |

S2. What is your age?

_____ years old

o I prefer not to answer

**PN: TERMINATE IF <18 YEARS OR PREFER NOT TO ANSWER.**

S3a. Please indicate if you currently have any chronic health condition – that is, any long-term condition that has been diagnosed by a health professional and that has lasted or is expected to last 6 months or longer.

| Yes, I currently have at least one chronic health condition that has been diagnosed by a healthcare professional | 1 |
| --- | --- |
| No, I do not currently have any chronic health condition that has been diagnosed by a healthcare professional | 2 |
| I don’t know | 99 |

**PN: QUOTAS ARE AS FOLLOWS:**

- 18-64 year olds – n= 250 with chronic condition
- 65-75 years – n=240 with chronic condition
- 76+ years – n = 160 with chronic condition

S3b. In the last year, about how many times have you done the following?

*Please do not count times when you were an overnight patient in a hospital.*

|  |  | I don’t know |
| --- | --- | --- |
| Gone to an emergency room | __# | o |
| Filled a prescription | __# | o |
| Had a lab test (e.g. blood test, urine test, etc.) | __# | o |
| Had an X-Ray, MRI, or CT/CAT Scan | __# | o |
| Visited your regular doctor or place of care | __# | o |
| Visited a specialist | __# | o |

**PN: RANDOMIZE. RANGE 0-99**

S4. In which province do you live?

|  |  | **QUOTAS ADULTS 18-64 YRS** | **QUOTA SENIORS 65-74 YRS** | **QUOTA SENIORS 76+ YRS** |
| --- | --- | --- | --- | --- |
| British Columbia | 1 | **55-75** | **35-55** | **15-35** |
| Alberta | 2 | **50-70** | **30-50** | **15-35** |
| Saskatchewan | 3 |  | **15-30** |  |
| Manitoba | 4 | **25-45** |  | **10-20** |
| Ontario | 5 | **190-215** | **110-135** | **65-95** |
| Quebec | 6 | **100 - 130** | **60-80** | **35-55** |
| New Brunswick | 7 |  |  |  |
| Nova Scotia | 8 |  |  |  |
| Prince Edward Island | 9 | **25 - 35** | **15-25** | **8-15** |
| Newfoundland | 10 |  |  |  |
|  |  | **500** | **300** | **200** |

**MAIN QUESTIONNAIRE**

**SECTION A: PATIENT CHARACTERISTICS / PRIVACY EXEPERIENCES [DO NOT SHOW SECTION TITLES]**

A1a. In the past year, have you gone online or used mobile apps for any of the following? *Please select all that apply.*

**PN: RANDOMIZE. ANCHOR NONE OF THE ABOVE AT BOTTOM.**

| Online Banking | 1 |
| --- | --- |
| Online Shopping | 2 |
| Managing household utilities (paying bills, etc.) | 3 |
| Email | 4 |
| General searching on the internet | 5 |
| Seeking medical information on the internet | 6 |
| Other health-related purposes | 7 |
| Watching videos/movies online | 8 |
| Online Surveys | 9 |
| Texting via mobile device or tablet | 10 |
| Playing games online (mobile devices, websites) | 11 |
| Using Apps on mobile device or tablet | 12 |
| Video messaging (Facetime, Skype, etc.) | 13 |
| Downloading music or podcasts online | 14 |
| None of the above | 99 |

**ASK IF SELECTED CODE 6 OR AT A1a**

A1b. Which of the following **HEALTH-RELATED online / mobile app tasks** have you performed in the past 12 months? *Please select all that apply.*

**PN: RANDOMIZE. ANCHOR NONE OF THE ABOVE AT BOTTOM.**

| Prescription renewal | 1 |
| --- | --- |
| Accessed your personal medical records | 2 |
| Made an appointment with a healthcare provider | 3 |
| Emailed healthcare provider (e.g. about a health issue / concern) | 4 |
| Virtual visit to healthcare provider | 5 |
| Accessed personal lab results | 6 |
| Accessed personal vaccine records | 7 |
| Accessed your personal prescription history | 8 |
| Used / participated in a social network service | 9 |
| Rated a healthcare provider online (e.g. RateMDs.com) | 10 |
| Looked up health information | 11 |
| Tracked / monitored personal health | 12 |
| Used self-diagnosing tools | 13 |
| None of the above | 99 |

**ASK ALL**

A2. How would you rate **your level of involvement** with how you manage your health in partnership with your healthcare providers?

| Very involved | 1 |
| --- | --- |
| Somewhat involved | 2 |
| Not very involved | 3 |
| Not at all involved | 4 |
| I don’t know | 99 |

A3. How would you rate your **confidence participating in your healthcare** with your healthcare providers?

| Very confident | 1 |
| --- | --- |
| Somewhat confident | 2 |
| Not very confident | 3 |
| Not at all confident | 4 |
| I don’t know | 99 |

A4. Overall, how would you rate the **quality of healthcare** you received in the **last 12 months**?

| Excellent | 1 |
| --- | --- |
| Good | 2 |
| Fair | 3 |
| Poor | 4 |
| I don’t know | 99 |

**PROGRAMMING NOTE: Program a gradient 4-point scale. Scale headers = Very sensitive, Somewhat sensitive, Not very sensitive, Not at all sensitive. RANDOMIZE. ALLOW ‘I don’t know’ OPTION.**

A5a. In your opinion, how **sensitive** are each of the following types of **medical records** *(i.e. medical information stored by a healthcare professional, such as your doctor, in an electronic health record)*?

*Please use a scale of 1 to 4 where 1 is ‘not at all sensitive’ and 4 is ‘very sensitive’.*

1. Contact and demographic information (e.g. Name, age, address)
2. Medications
3. Test results (e.g. lab results)
4. Past medical history
5. History of substance abuse
6. Mental health records
7. Sexual health records
8. Domestic violence
9. Genetic data
10. Psychotherapy notes (i.e. notes recorded during a counseling session.

**PROGRAMMING NOTE: Program a gradient 4-point scale. Scale headers = Very sensitive, Somewhat sensitive, Not very sensitive, Not at all sensitive. RANDOMIZE. ALLOW ‘I don’t know’ OPTION.**

A5b. And, in your opinion, how **sensitive** are each of the following types of **personal data** *(i.e. information you have personally tracked or recorded, for example on a mobile health app or online program)*?

*Please use a scale of 1 to 4 where 1 is ‘not at all sensitive’ and 4 is ‘very sensitive’.*

1. Vital signs (e.g. Heart rate, blood pressure)
2. Stress levels
3. Mood tracking
4. Physical activity
5. Weight
6. Diet
7. Blood levels (e.g. A1C for diabetic patients)
8. Medication
9. Medication or treatment adherence
10. Sleep patterns
11. Smoking
12. Alcohol consumption
13. Environmental exposure (e.g. tracking physical / social locations associated with physical and/or mental health)
14. Meditation and mindfulness exercises
15. Personal genetic test
16. Menstrual cycle **[SHOW FEMALES ONLY]**
17. Contact and demographic information (e.g. name, age, address)
18. Location / region
19. Communication logs (i.e. any correspondence with other users, App or online program provider, or healthcare providers within the App or online program)

A6a. Overall, how would you rate your **level of privacy** in your past **online experiences** (e.g. internet, mobile applications)?

| Excellent | 1 |
| --- | --- |
| Good | 2 |
| Fair | 3 |
| Poor | 4 |
| I don’t know | 99 |

A6b. Have you or a member of your family ever experienced a breach where your personal health information was used inappropriately or released without your consent?

| Yes | 1 |
| --- | --- |
| No | 2 |
| I don’t know | 99 |

**PN: ‘No’ & ‘I don’t know’ ARE MUTUALLY EXCLUSIVE**

**ASK A6c/d IF YES (CODE 1) AT A6b**

A6c. Who experienced a breach? *Please select all that apply.*

| I personally experienced a breach | 1 |
| --- | --- |
| My family member experienced a breach | 2 |
| I don’t know | 99 |

A6d. Was the breach resolved?

| Yes, it was resolved to my satisfaction | 1 |
| --- | --- |
| Yes, it was resolved but not to my satisfaction | 2 |
| No, it was not resolved | 3 |
| I don’t know | 99 |

A7a. Overall, how would you rate your privacy experiences in your past **healthcare** encounters?

| Excellent | 1 |
| --- | --- |
| Good | 2 |
| Fair | 3 |
| Poor | 4 |
| I don’t know | 99 |

**SHOW A7c/d ON SAME SCREEN AFTER SELECTING RESPONSE AT A7a**

**ASK IF SELECTED CODES 1-4 AT A7a**

A7b. Why do you rate your privacy experiences in your past healthcare encounters as ______ **[PIPE IN SELECTION AT A7b]**? *Please be as descriptive as possible.*

|  |
| --- |

**ASK IF SELECTED CODE 99 AT A7b**

A7c. Why do you say that? *Please be as descriptive as possible.*

|  |
| --- |

A7d. To what extent do you feel patients’ healthcare records held by healthcare professionals in Canada are kept private / confidential?

| Very private | 1 |
| --- | --- |
| Somewhat private | 2 |
| Not very private | 3 |
| Not at all private | 4 |
| I don’t know | 99 |

A7e. Have you or a family member experienced a **healthcare-related privacy breach** in the past?

| Yes | 1 |
| --- | --- |
| No | 2 |
| I don’t know | 99 |

**PN: ‘No’ & ‘I don’t know’ ARE MUTUALLY EXCLUSIVE**

**ASK A7f/g IF YES (CODE 1) AT A7e**

A7f. Who experienced a healthcare-related breach? *Please select all that apply.*

| I personally experienced a breach | 1 |
| --- | --- |
| My family member experienced a breach | 2 |
| I don’t know | 99 |

A7g. Was the breach resolved?

| Yes, it was resolved to my satisfaction | 1 |
| --- | --- |
| Yes, it was resolved but not to my satisfaction | 2 |
| No, it was not resolved | 3 |
| I don’t know | 99 |

**SECTION B: INTENT TO USE ACCESS**

**VIGNETTE 1: PERCEPTIONS ABOUT ACCESS HEALTH [PN: DO NOT SHOW TITLE]**


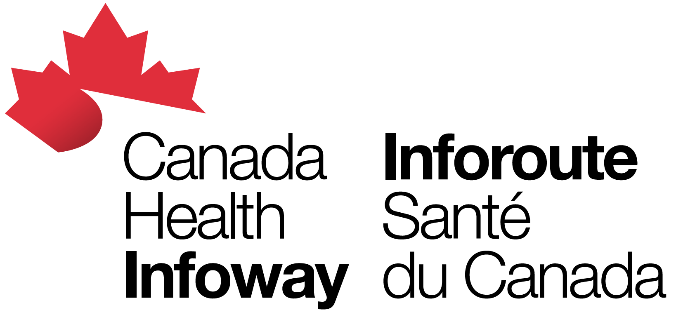


Canada Health Infoway is a federally funded, independent, not-for-profit organization, who works to implement the use of digital health solutions across Canada.

Canada Health Infoway, with funding from Health Canada, has a program called ACCESS Health that is providing Canadians with access to their personal health information and to digitally-enabled health services. The program aims to provide you as a Canadian with a greater ability to manage your care, more convenience and increased control over your personal health information.

One part of the program is a digital platform that Canada Health Infoway is building, called the ACCESS Gateway. Available to every Canadian, once built, the service will allow you do things like:

- View your personal health information online,
- Share your health information with others (e.g. friends/family, health care professionals like your doctor, or commercial service providers such as health tracking/monitoring apps)
- electronically book medical appointments,
- securely communicate with care providers, and
- use self-management applications.

**Canada Health Infoway is engaging with Canadians to better understand their needs, priorities, and expectations in order to guide the development of the ACCESS Gateway.**

**SHOW ON NEW SCREEN**

B1a. Based on your understanding, would you register for the ACCESS Gateway?

| Very likely | 1 |
| --- | --- |
| Somewhat likely | 2 |
| Not very likely | 3 |
| Not at all likely | 4 |
| I don’t know | 99 |

**SHOW ON SAME SCREEN**

B1b. Why do you say that? *Please be as descriptive as possible.*

|  |
| --- |

**VIGNETTE 1b : PERCEPTIONS ABOUT ACCESS GATEWAY [DO NOT SHOW TITLE]**

Canada Health Infoway's "Trust Framework" is the go-to guide for what participants in shared digital health systems (such as the ACCESS Health Gateway) need in order for health information systems to be able to connect and share with each other in the way that meets the needs and expectations of Canadians.

It includes rules for operation and participation, such as policies and agreements around data sharing and how users can control their health information. This allows users to access multiple systems and data sources securely and easily with fewer credentials (e.g. avoid multiple usernames and passwords), via the ACCESS Gateway.

Individuals will be able to control access to their health information (i.e. allowing access of your data to certain individuals – for example, friends/family, healthcare providers, commercial service providers) in a secure manner. This lets individuals control the access to personal health information by enabling/disabling it for various mobile apps / website applications from one place.

**SHOW B2 ON NEW SCREEN**

B2. To what extent would the following safeguards have an **impact** on your **overall comfort levels** with registering and using ACCESS Gateway?

**PN: RANDOMIZE**

|  | Significant impact | Some impact | Not much impact | No impact at all | I don’t know |
| --- | --- | --- | --- | --- | --- |
| Ability to access health records online | 4 | 3 | 2 | 1 | 99 |
| Ability to hide or mask sensitive information from those you have given access | 4 | 3 | 2 | 1 | 99 |
| Regular privacy and security audits of ACCESS Gateway | 4 | 3 | 2 | 1 | 99 |
| Text or email notification when a healthcare provider accesses my records | 4 | 3 | 2 | 1 | 99 |
| Ability to view a history of who accessed my records and when | 4 | 3 | 2 | 1 | 99 |
| Knowing I would be informed of any privacy and security breaches that may have occurred with my health records | 4 | 3 | 2 | 1 | 99 |
| More knowledge about the policy and procedures that protect my health records | 4 | 3 | 2 | 1 | 99 |
| A strict policy that my health information could not be sold or released to any organization/business that was not a part of the healthcare system | 4 | 3 | 2 | 1 | 99 |

B3a. Based on your understanding of the “Trust Framework” and the availability of the safeguards listed in the previous question, would you register and use the ACCESS Gateway?

| Very likely | 1 |
| --- | --- |
| Somewhat likely | 2 |
| Not very likely | 3 |
| Not at all likely | 4 |
| I don’t know | 99 |

**SHOW ON SAME SCREEN**

B3b. Why do you say that? *Please be as descriptive as possible.*

|  |
| --- |

B4. Please rate your level of agreement with each of the following statements.

**PN: RANDOMIZE**

|  | Strongly agree | Somewhat agree | Somewhat disagree | Strongly disagree | I don’t know |
| --- | --- | --- | --- | --- | --- |
| I believe that Canada Health Infoway will provide enough safeguards to make me feel comfortable using the ACCESS Gateway to access and manage who sees my personal health information | 4 | 3 | 2 | 1 | 99 |
| I feel assured that the legal and technological safeguards will adequately protect my data | 4 | 3 | 2 | 1 | 99 |
| I feel that encryption and other security advances make it safe for me to use the ACCESS Gateway | 4 | 3 | 2 | 1 | 99 |
| In general, the electronic sharing of my health information within the healthcare system is safe and secure | 4 | 3 | 2 | 1 | 99 |

B5. What questions do you have about ACCESS Gateway that would increase your confidence, or interest in participating?

|  |
| --- |

**SECTION C: ACCESS CONSENT PREFERENCES**

**ASK SECTION C IF SELECTED CODES 1 OR 2 AT EITHER B1a OR B3a. IF SELECTED CODES 3, 4, OR 99 AT BOTH, GO TO SECTION D.**

SHOW ON SEPARATE SCREEN: ***The next series of questions will provide scenarios around the use of ACCESS Gateway. Please answer them to the best of your ability based on the information provided.***

**VIGNETTE 2 : CONSENT MANAGEMENT [DO NOT SHOW TITLE]**

The ACCESS Gateway will include consent technology that will manage individual’s preferences for sharing their health information. For example, an individual may use a healthcare application, that could be a mobile app or web application, which provides access to their personal health information. Health data may include, but is not limited to:

- patient summary (e.g., history of diagnoses, investigations, treatments, care plan)
- medication history
- lab results
- diagnostic reports (e.g., medical imaging)
- data from digital services and tools

If the healthcare application is connected to the ACCESS Gateway, an individual will be able to access their personal health information in one place through the ACCESS Gateway and put preference in place for who can access their personal health information.

**SHOW C1 ON NEW SCREEN**

C1. Through the ACCESS Gateway, you may set preferences for what applications can access your personal health information.

*Data sources may include medical history from clinics and hospitals; lab records; clinical and diagnostic imaging; drug and pharmacy services; lab testing, and data from digital services and tools.*

Which of the following would you prefer? *Please note that you would be able to opt-out of data sources at any point.*

*Please select one only.*

| Provide consent where you opt-in to have **ALL** health care applications access **ALL** of the personal health information linked to your account. | 1 |
| --- | --- |
| Provide consent where you opt-in to have **SPECIFIC** applications access **ALL** the personal health information linked to your account. | 2 |
| Provide consent to have **All** applications access **SPECIFIC** sources of personal health information linked to your account. | 3 |
| Provide consent where you Opt-in to have **SPECIFIC** applications access **SPECIFIC** sources of personal health information linked to your account. | 4 |
| No preference / I don’t know | 99 |

**VIGNETTE 2 : CONSENT MANAGEMENT [DO NOT SHOW TITLE]**

The ACCESS Gateway may provide ways to allow patients to authorize or delegate access to their personal health information to:

- healthcare providers,
- verified members (such as family and friends),
- digital services and tools they have chosen to use.

**SHOW C2 ON NEW SCREEN**

C2. You have a partner, friend, or family member who supports you in managing your health. You are considering authorizing them to access your personal health information available through ACCESS Gateway. To do so, you must provide consent to grant them access to your records.

  What types of information do you require in order to make an informed decision? *Please select all that apply.*

**PN: RANDOMIZE. KEEP CODES 3 & 4 TOGETHER. ANCHOR CODE 98, 9, & 99 AT BOTTOM.**

| Type(s) of information they can access | 1 |
| --- | --- |
| What they can do with your data | 2 |
| Potential risks of granting them access | 3 |
| Potential benefits of granting them access | 4 |
| Contact information for privacy questions and concerns | 5 |
| Contact information for making privacy complaints | 6 |
| ACCESS Gateway functions that allow you to monitor activity | 7 |
| How to restrict others’ access to your personal health information | 8 |
| How to revoke others’ access to your personal health information | 9 |
| Other – please specify ______ | 98 |
| None of the above | 10 |
| I do not intend on sharing information with them | 99 |

**PN: CODES 9 & 99 ARE MUTUTALLY EXCLUSIVE**

C3. You are interested in enrolling in a digital service that is available from a commercial service provider (i.e. a company storing medical information that allows you to manage your health). The digital tool requires access to your lab results to allow you to track your blood work and provide you with curated content for your self-management. To use the tool, you must provide consent to grant it access to your records.

What types of information do you require in order to make an informed decision? *Please select all that apply.*

| Type(s) of information the digital service can access | 1 |
| --- | --- |
| What the digital service can do with your data | 2 |
| Potential risks of granting access | 3 |
| Potential benefits of granting access | 4 |
| How to ask more questions around information sharing / privacy | 5 |
| How to file complaints around how information is shared | 6 |
| ACCESS Gateway functions that allow you to monitor activity | 7 |
| Types of data access controls available (e.g. ways that the digital service could provide consent for others to access your information) | 8 |
| How to revoke access | 9 |
| Other – please specify ______ | 98 |
| None of the above | 10 |
| I do not intend on sharing information with them | 99 |

C4. You have read the online consent form and are comfortable with the information provided. You are ready to consent to share your records with others you have given access to (e.g. friends/family, healthcare providers, companies storing health data such as an App).

What would be your preferred method of agreeing to opt-in? *Please select one only.*

| Clicking on a ‘yes’ button on the online form | 1 |
| --- | --- |
| Providing a digital signature | 2 |
| Entering your log-in information | 3 |
| Using another credential to authenticate (e.g. using the same sign-in information you use for other online services) | 4 |
| Verification through another device (such as a smartphone or tablet) | 5 |
| Other – please specify ________ | 99 |

**VIGNETTE 2 : CONSENT MANAGEMENT [DO NOT SHOW TITLE]**

The ACCESS Gateway will also allow individuals to securely control access to their information via consent directives (i.e. allowing or restricting access of your data to certain individuals – for example, friends/family, healthcare providers, commercial service providers).

Consent directives gives individuals the option to block or restrict access to their personal health information.

**SHOW C5 ON NEW SCREEN**

C5. When sharing personal health information, people have control over their privacy preferences (e.g., who can see the personal health information, why they can see it, which parts they can see, etc.).

How important is the ability to set and change your privacy preferences for sharing your personal health information?

| Very important | 1 |
| --- | --- |
| Somewhat important | 2 |
| Not very important | 3 |
| Not at all important | 4 |
| I don’t know | 99 |

C6a. If the ACCESS Gateway allows you to consent to share either “all” or “none” of the personal health information from a specific source* with those individuals or groups you granted access.

**Data sources include: medical history from clinics and hospitals; lab records; clinical and diagnostic imaging; drug and pharmacy services; lab testing, and e-services data.*

What are your feelings about this method for giving your consent to share your personal health information? *Please select one only.*

| The consent process reflects my needs | 1 |
| --- | --- |
| The consent process does **not** reflect my needs – I would like more choices | 2 |
| I don’t know | 99 |

**ASK IF SELECTED CODE 2 AT C6a**

C6b. You mentioned that you would like more choices in how you can consent to share your personal health information from a specific source with those you granted access. What other ways would you like to limit your personal health information? *Please be as descriptive as possible.*

|  |
| --- |

 C7. A healthcare clinic where you receive care started making their records available through a digital tool. You are considering enrolling in this digital service so that you can have access to a more comprehensive set of your personal health information.

Given you have the ability to apply consent directives (*consent directives give you the option to restrict access to your personal health information)* in the future, who would you want to be allowed to access this new source of personal health information by default? *Please select all that apply.*

| **Your Healthcare Providers –** those who provide direct care to you (e.g. doctors, nurses, specialists, therapists) | 1 |
| --- | --- |
| **Your Authorized Members** – those you granted access to your personal health information | 2 |
| **Your digital services & tools** – that you have previously authorized to access your personal health information | 3 |
| **None of the above** – I would like to grant access individually or to each group | 99 |

**PN: CODE 99 MUTUALLY EXCLUSIVE**

C8. The ACCESS Gateway will have a notification system which will provide prompts related to consent and access to your personal health information or digital services.

  For each of the following notifications, select which is the best method to notify you. *Please select one method of notification for each. If you would like the same notification for all actions, please only select one at ‘All of the above’.*

|  | ACCESS Gateway  (i.e. within the online dashboard or inbox) | Email | SMS / Text message | Phone call | Other – please specify |
| --- | --- | --- | --- | --- | --- |
| Request for personal health information or digital service /tool data | 1 | 2 | 3 | 4 | 5 |
| Authorized party has accessedpersonal health information or digital service /tool data | 1 | 2 | 3 | 4 | 5 |
| Authorized party has updated personal health information or digital service /tool data | 1 | 2 | 3 | 4 | 5 |
| ACCESS Gateway was accessed on an unfamiliar device | 1 | 2 | 3 | 4 | 5 |
| Privacy policy update | 1 | 2 | 3 | 4 | 5 |
| Updated consent notices from digital service /tool (i.e. when they change consent policies) | 1 | 2 | 3 | 4 | 5 |
| Consent directive is about to expire | 1 | 2 | 3 | 4 | 5 |
| Healthcare provider override  (e.g. ability for doctor to access restricted data) | 1 | 2 | 3 | 4 | 5 |
| Privacy violation | 1 | 2 | 3 | 4 | 5 |
| All of the above | o | o | o | o | o |

C9. In addition to receiving notifications, ACCESS Gateway will allow users to audit and summarize consent directives *(i.e. allowing or restricting access of your data to certain individuals – for example, friends/family, healthcare providers, commercial service providers).*

Please rate the usefulness of each of the following types of consent reporting functions:

|  | Very useful | Somewhat useful | Not very useful | Not at all useful | I don’t know |
| --- | --- | --- | --- | --- | --- |
| Delegates that have consent to allow third parties to access your data on your behalf | 4 | 3 | 2 | 1 | 99 |
| Who was granted consent (i.e. who you have provided consent to access your data) | 4 | 3 | 2 | 1 | 99 |
| When consent was granted | 4 | 3 | 2 | 1 | 99 |
| Temporary consents applied and expiry dates | 4 | 3 | 2 | 1 | 99 |
| Summary of information each delegate has accessed | 4 | 3 | 2 | 1 | 99 |
| Records of revoked consent | 4 | 3 | 2 | 1 | 99 |
| A summary report of all data above | 4 | 3 | 2 | 1 | 99 |

**SECTION D: RESPONDENT DEMOGRAPHICS**

D1. What is your ethnicity? *Please select all that apply.*

| South Asian (e.g. East Indian, Pakistani, Sri Lankan, etc) | 1 |
| --- | --- |
| Southeast Asian (e.g. Vietnamese, Cambodian, Malaysian, etc.) | 2 |
| West Asian (e.g. Iranian, Afghan etc.) | 3 |
| White/Caucasian | 4 |
| Indigenous | 5 |
| Black | 6 |
| Latin American | 7 |
| Chinese | 8 |
| Korean | 9 |
| Japanese | 10 |
| Other (specify) ___________ | 11 |
| I prefer not to answer | 99 |

D2. What is your current marital status? *Please select the one that best describes you.*

| Single | 1 |
| --- | --- |
| Married | 2 |
| Common law / living with partner | 3 |
| Separated | 4 |
| Divorced | 5 |
| Widowed | 6 |
| I prefer not to answer | 99 |

D3. Are you currently a caregiver to a family member or friend, or do you have a family member of friend who acts as an informal caregiver to you?

*A caregiver is an individual who provides ongoing care and assistance, without pay, for family members and friends in need of support due to physical, cognitive, or mental conditions.*

*Please select all that apply.*

| I provide care to a family member / friend | 1 |
| --- | --- |
| A family member / friend provides care for me | 2 |
| None of the above | 99 |

D4. What best describes the area you currently live? *Please select one only.*

| Rural (population of less than 50,000) | 1 |
| --- | --- |
| Small town (population between 50,000 – 250,000) | 2 |
| Large city (population from 250,000 – 1 million) | 3 |
| Metropolitan (population of 1 million or more) | 4 |
| I prefer not to answer | 99 |

D5. What is the last year of education that you have completed? *Please select one only.*

| Elementary (7 years or less) | 1 |
| --- | --- |
| High school, general or vocational (8 to 12 years) | 2 |
| College (pre-university, technical training, certificate, accreditation) | 3 |
| University certificates and diplomas | 4 |
| University Bachelor degree | 5 |
| University Master's degree | 6 |
| University Doctorate (PhD) | 7 |
| I prefer not to answer | 99 |

D6. What is your current employment status? *Please select one only.*

| Working full-time | 1 |
| --- | --- |
| Working part-time | 2 |
| Homemaker, no outside employment | 3 |
| Student | 4 |
| Unemployed | 5 |
| Retired | 6 |
| Disabled | 7 |
| Other | 98 |
| I prefer not to answer | 99 |

D7. Which of the following categories best reflects your total annual household income? *Please select one only.*

| Less than $20,000 | 1 |
| --- | --- |
| $20,000 to $39,999 | 2 |
| $40,000 to $59,999 | 3 |
| $60,000 to $79,999 | 4 |
| $80,000 to $99,999 | 5 |
| $100,000 to $119,999 | 6 |
| $120,000 or more | 7 |
| I prefer not to answer | 99 |

D8. Which of the following best describes the type of health insurance coverage you currently have? *Please select one only.*

| Public / provincial coverage only | 1 |
| --- | --- |
| Private insurance | 2 |
| No coverage | 3 |
| I don’t know | 9 |
| I prefer not to answer | 99 |

**NEXT SCREEN:**

**This concludes our survey. Thank you very much for your participation.**
